# Contextual and culturally adapted interventions to improve HIV outcomes: a scoping review

**DOI:** 10.1186/s12879-025-12143-3

**Published:** 2025-12-29

**Authors:** Witdiawati Witdiawati, Kusman Ibrahim, Neti Juniarti, Dadang Purnama, Laili Rahayuwati

**Affiliations:** 1 Doctoral Program, Faculty of Medicine, Universitas Padjadjaran, Sumedang, West Java 45363 Indonesia; 2 Department of Community Nursing, Faculty of Nursing, Universitas Padjadjaran, Sumedang, West Java 45363 Indonesia; 3 Department of Medical-Surgical Nursing, Faculty of Nursing, Universitas Padjadjaran, Sumedang, West Java 45363 Indonesia

**Keywords:** Antiretroviral therapy, Cultural adaptation, Contextual intervention, Human immunodeficiency virus

## Abstract

**Background:**

Antiretroviral therapy is widely available, yet structural, social, and cultural barriers still limit human immunodeficiency virus prevention and care. Prevention tools such as consistent condom use and pre-exposure prophylaxis (PrEP) remain essential. Interventions that ignore local context and culture often have low uptake, especially among adolescents, Indigenous communities, and sexual minorities.

**Objective:**

To map and synthesize contextually and culturally adapted interventions that aim to improve outcomes related to human immunodeficiency virus.

**Method:**

This scoping review followed the Arksey and O Malley framework and the PRISMA ScR guideline. We searched Scopus, PubMed, and CINAHL used keywords for human immunodeficiency virus, contextual delivery, and cultural adaptation. Eligible studies involved people living with human immunodeficiency virus or populations at substantial risk. Interventions had to be tailored to setting or culture in health care or community contexts. We defined contextual or cultural adaptation as development or modification with local stakeholder input and the inclusion of culturally salient elements such as values, norms, language, imagery, community delivery agents, or locally meaningful practices. As a scoping review, we did not conduct formal study-level quality appraisal. Data were extracted with a standardized form and synthesized narratively.

**Results:**

Eighteen studies met the inclusion criteria. Interventions included community-based counseling and peer programs; digital approaches such as mobile and electronic health (including short message service reminders, smartphone applications, and tele-counseling); complementary mind–body strategies; and couple or family models tailored for marginalized groups. Seven cross-cutting themes appeared: (1) culturally grounded psychosocial support; (2) technology-enabled adherence and care engagement; (3) mind–body strategies addressing stigma, stress, and resilience; (4) couple and family involvement for communication and support; (5) tailoring for adolescents and structurally marginalized groups; (6) addressing specific sexual behaviours and HIV prevention (e.g., reducing condomless sex, increasing HIV testing, and uptake of PrEP and post-exposure prophylaxis [PEP]); and (7) quality-of-life and mental-health enhancement. Many studies reported improvements associated with these approaches, such as better adherence, lower stigma, increased HIV testing and PrEP uptake, and reductions in condomless sex, alongside gains in psychological resilience and mental health. Effects on clinical endpoints were mixed, and some studies reported null findings.

**Conclusions:**

Contextual and cultural adaptation is a promising direction for improving engagement with human immunodeficiency virus prevention and care. Future research should use adequately powered evaluations in diverse settings, assess durability and scalability in routine systems, and report adaptation processes in enough detail to support replication.

**Supplementary Information:**

The online version contains supplementary material available at 10.1186/s12879-025-12143-3.

## Introduction

HIV and AIDS remains a pressing global public health issue, with an estimated 39 million people living with HIV worldwide in 2022 [1]. Despite progress in expanding access to antiretroviral therapy (ART), HIV control success still shows significant disparities between countries and populations, particularly in resource-limited regions like Sub-Saharan Africa. Recent reports indicate that global progress toward the 95-95-95 targets remains uneven, with notable gaps in treatment retention and viral suppression [2]. The main obstacles are not only the availability of ART, but also issues of uptake, treatment continuity, and patient adherence, which are influenced by stigma, social pressure, poverty, and limitations in the health care system [3]. This highlights the importance of approaches that can directly address barriers related to stigma and social exclusion, while acknowledging that poverty and health system constraints require broader policy and structural solutions.

Although HIV/AIDS has long been a global health priority, inequalities in service availability, low health system capacity, and poor integration with primary health care mean that many individuals still do not receive early diagnosis, do not initiate ART in a timely manner, or discontinue treatment [4]. In many low- and middle-income countries, access is further hindered by geographic barriers, hidden costs, and a lack of trained health workers. These barriers demonstrate that effective responses must go beyond pharmacological solutions and incorporate strategies that account for psychosocial, cultural, and structural dimensions [5].

The success of ART is strongly influenced by non-medical factors, including stigma within families, communities, and health facilities, distrust of formal health services, and cultural norms that may restrict access to sexual and reproductive health information, especially for women, adolescents, and conservative communities [6]. Marginalized groups such as people who inject drugs, sex workers, LGBTQ + communities, and indigenous peoples face dual challenges of social exclusion and lack of culturally sensitive services [7]. Together, these factors illustrate the need for contextual and culturally adapted interventions that specifically target engagement and adherence.

Over the past two decades, non-pharmacological interventions have rapidly evolved as adjuncts to ART in efforts to improve treatment outcomes. These interventions include individual or group counseling, community-based interventions and peer support, the use of digital technology such as eHealth and mHealth, and complementary therapies such as yoga, meditation, or touch therapy [8–10]. A growing body of research emphasizes the importance of integrating cultural aspects, local values, and socioeconomic conditions into intervention design to ensure contextualization and acceptability [11, 12]. Examples of successful culturally adapted approaches include adherence clubs in South Africa and community ART groups in Mozambique, which improved retention and viral suppression by embedding ART delivery within peer and community structures [12, 13], and the Mothers2Mothers program, which uses peer mentors rooted in local motherhood norms to strengthen engagement in prevention of mother-to-child transmission services [3, 14].

Prior reviews of culturally adapted HIV interventions exist, including an early synthesis of culturally grounded prevention strategies [15] and a recent scoping review focused on Black or African American populations [16], yet most are limited in scope, time frame, or specific population focus, underscoring the need for a broader and updated map of approaches across populations and outcomes. For the purposes of this review, culturally sensitive services are defined as interventions developed or adapted with direct community input, embedding cultural elements such as values, norms, language, imagery, trusted peer delivery agents, or local governance structures, and going beyond mere translation of materials. These services may include psychosocial counseling, community-based peer support, technology-enabled supports such as mHealth, and family or couple-centered programs. Importantly, they are not synonymous with non-pharmacological interventions, but instead represent a cross-cutting approach that can complement biomedical strategies. Building on this rationale, the objective of this review is to identify, categorize, and synthesize contextual and culturally adapted interventions aimed at improving ART adherence, reducing stigma and risky behaviors, and enhancing quality of life and service engagement.

## Method

### Study design

This study used a scoping review design based on the Arksey and O Malley framework from 2005 which is suitable for mapping broad and heterogeneous evidence [17, 18]. A scoping review was selected because the aim was to comprehensively map contextual and culturally adaptive interventions that improve health outcomes among people living with HIV and to identify gaps in the evidence base. A systematic review with meta analysis was not undertaken due to substantial heterogeneity in intervention types settings comparators and outcome measures which would limit the interpretability of pooled estimates and this rationale is reiterated in the limitations. Following PRISMA-ScR guidance, we did not undertake any formal risk-of-bias or other study-level quality appraisal, as scoping reviews prioritize mapping the breadth of evidence over formal quality judgments. The review proceeded in five stages which were identifying the research question identifying relevant studies selecting studies by prespecified criteria extracting data and analyzing reporting and narratively presenting findings.

### Search strategy and eligibility criteria

The database search was conducted and last updated on 31 March 2025. Three databases were used namely Scopus, PubMed, and CINAHL because of their coverage indexing quality and relevance to public health and intervention research. Screening and duplicate handling used Rayyan with automated duplicate detection followed by manual verification. The full database-specific search strings are provided in Appendix 1 to support replication.

Search terms combined keywords and MeSH terms using Boolean operators. To minimize missed retrievals we used a two-tier strategy. Tier one covered HIV and intervention concepts while tier two covered culture context and delivery concepts that were applied flexibly during screening rather than strictly at query time. Illustrative search terms included HIV or human immunodeficiency virus combined with intervention, program, approach, trial, evaluation, implementation, service, delivery, or model. These were further combined with terms such as cultural adaptation, cultural tailoring, culturally adapted, cultural competence, cultural safety, Indigenous led, community led, community driven, community based, peer led, context specific, locally adapted, place based, structural intervention, stigma reduction, adherence support, or differentiated service delivery. Searches were optionally filtered with outcome related terms, including adherence, stigma, engagement in care, retention, linkage, viral suppression, or quality of life. Consistent with methodological cautions that narrow adaptation terms can under retrieve we also screened broadly worded HIV intervention studies at full text to identify embedded cultural or contextual components even when these were not indexed in titles abstracts or keywords.

**Fig. 1:**
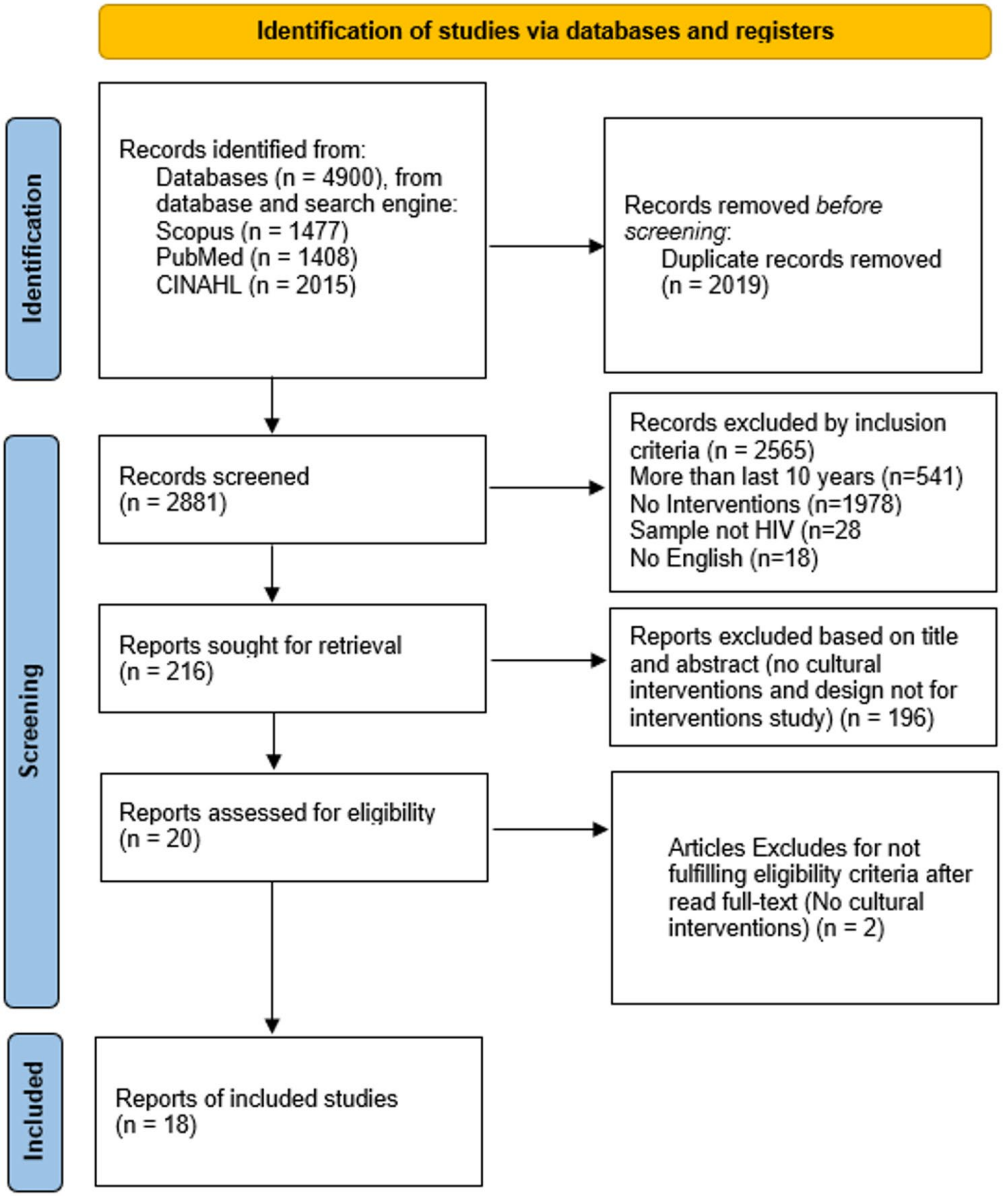
PRISMA flow diagram

Two reviewers independently screened titles and abstracts then full texts in Rayyan with disagreements resolved by discussion and when required by a third reviewer. The PRISMA flow diagram specifies reasons for full text exclusion which include wrong population wrong study type absence of cultural or contextual elements and outcomes not relevant (Fig. 1) [19]. To address potential under retrieval from narrow adaptation terms we prespecified a robustness step that allowed identification of cultural or contextual adaptation during full text screening even when adaptation terms were absent from indexing. We also specified a feasibility subset screen that removed adaptation terms to gauge whether additional eligible studies would surface and we describe this consideration in the limitations.

### Inclusion and exclusion criteria

Eligibility followed the PCC framework. The population was people living with HIV and people first language is used throughout in line with BHIVA People First guidance. The concept was non-pharmacological interventions that were contextual or culturally adapted defined operationally as strategies that explicitly or implicitly integrate local norms beliefs practices languages or community engagement into design delivery or implementation for example peer or indigenous led models co design with community stakeholders place based delivery or materials tailored to cultural practices. The context included health service settings community programs and other relevant settings in any country. Inclusion criteria were original peer reviewed articles studies about implementation or feasibility of interventions with contextual or cultural adaptation identified through full text assessment even when not labeled as such in indexing interventions aiming to improve adherence, stigma, quality of life, engagement retention linkage or viral suppression and publication in English within 1 January 2016 to 31 March 2025. Eligible designs included randomized and non-randomized evaluations and peer reviewed protocols that described adaptation features and a planned evaluation. Protocols were included to map the landscape of approaches and were reported separately from completed evaluations.

Exclusion criteria were purely pharmacological interventions without socio cultural components abstract only studies articles not relevant to HIV interventions and editorials commentaries or conference reports. Pharmacological delivery approaches were eligible when the delivery itself incorporated contextual or cultural adaptation for example antiretroviral therapy provided in culturally significant venues or through community led differentiated service delivery.

### Data extraction

A piloted standardized extraction form captured citation details study design and setting population characteristics intervention description including theoretical basis components delivery agents intensity duration setting cultural or contextual adaptation features and implementation strategies comparator where applicable outcomes and measures with timing effect estimates when reported implementation outcomes such as acceptability feasibility fidelity and reach and notes on equity and cultural safety. Two reviewers independently extracted data using Rayyan to organize records with a calibration exercise on a 10% subset before full extraction and disagreements resolved by discussion or by a third reviewer. Corresponding authors were contacted when essential information on adaptation features or outcomes was unclear.

### Data analysis

A reflexive thematic analysis was undertaken and findings are presented as a structured narrative synthesis. Steps included familiarization with extracted data independent open coding focused coding to derive subthemes mapping subthemes to higher order themes and iterative discussion to finalize the thematic framework. Studies were grouped by delivery strategy such as peer led community led or facility based by adaptation modality such as linguistic adaptation co design place based delivery or cultural safety training and by outcome domain.

## Results

### Study selection

A database search of CINAHL, PubMed, and Scopus identified 4,900 records. Duplicates were removed in Rayyan using automated detection and manual verification, leaving 2,881 records for screening. Title and abstract screening excluded 2,565 records, and 216 reports were sought for retrieval. Of these, 196 were excluded at title and abstract, and 20 reports were assessed for eligibility at full text. Two full text articles were excluded for not meeting the cultural intervention criterion, yielding 18 studies for synthesis. The time window was 1 January 2016 to 31 March 2025, and the language was English.

### Overview of included studies

The eighteen studies covered a range of culturally and contextually adapted approaches that addressed adherence, stigma, quality of life, mental health, HIV testing and PrEP uptake, reductions in condomless sex, and engagement in care. Designs included randomized trials, feasibility and pilot studies, and study protocols. Settings spanned Africa, Asia, North America, and Europe (Table 1). Interventions were delivered by peers, community agents, clinicians, or digital tools, and frequently integrated language tailoring, community co design, or delivery in community and culturally important sites. Across the eighteen included studies, seven recurring principles of cultural and contextual adaptation emerged. These principles cut across countries, designs, and intervention types and illustrate how programs became relevant and meaningful for people living with HIV.

**Table 1:** Extraction data

No	Authors, Year	Purpose	Country	Sample	Design	Interventions	Key Findings
1	(Bere et al. 2017)	To adapt and test the feasibility of a CBT-based intervention (Life-Steps) to suit the Zimbabwean cultural context to improve ART adherence.	Zimbabwe	42 adult HIV patients; 4 accompanying counselors	Feasibility study with a mixed-method approach	50-minute CBT session + 4 skills-based booster sessions (10–15 min); using the ADAPT-ITT model; implemented by non-specialist counselors	The Nzira Itsva intervention was feasible and acceptable, improving ART adherence (0% of 15 patients re-interviewed missed doses in the last 4 weeks, compared to 40% previously); counselors maintained fidelity (17–18/18) for 6 months.
2	(Li et al. 2017)	Evaluating the initial effectiveness of a resilience-based intervention (ChildCARE) in children affected by parental HIV/AIDS in China	China	790 children aged 6–17 years, and their primary caregivers	Randomized controlled trial, 4-arm cluster design	ChildCARE intervention: child level (10 sessions), caregiver (5 sessions), community (monthly activities); evaluation at 6 and 12 months	Child intervention increased resilience, support, and positive emotions (6 months); effects declined at 12 months. Child + caregiver intervention resulted in significant improvements in resilience, coping, hope, future control, and emotion regulation (12 months).
3	(Bogart et al. 2017)	Assessing the effectiveness of the “RISE” community-based intervention developed specifically for Black Americans in improving ART adherence.	United States of America	215 Black Americans HIV + participants; 107 intervention, 108 control	Randomized Controlled Trial (6 months), daily evaluation with electronic monitoring	RISE intervention: community-based counseling by trained peer counselors, using a problem-solving approach to cultural barriers (such as HIV stigma, medical mistrust) and referral to support services.	Adherence increased significantly: OR = 1.30 per month; cumulative effect after 6 months was large (OR = 4.76; Cohen’s d = 0.86); much larger than the effect of previous conventional HIV adherence interventions.
4	(Kurth et al. 2016)	Linguistically and culturally adapting the CARE + computer program for Spanish-speaking HIV patients and evaluating its effectiveness in supporting ART adherence and risk reduction	United States of America	494 Spanish-speaking HIV + patients, recruited from 3 clinics in NYC; 253 intervention, 241 control	A 12-month longitudinal RCT, with quantitative and qualitative evaluation.	Intervention: CARE + Spanish, a TAM (Technology Acceptance Model)-based computer counseling program, delivered during routine visits; includes ART, motivation, and risk reduction modules.	There was no statistically significant effect on ART adherence or viral load; however, the program was deemed highly culturally acceptable, systemically feasible, and low burden on clinical staff.
5	(Bremner et al. 2016)	Evaluating the effectiveness of Reiki with music compared to music alone in reducing stress, anxiety, pain, and depression in PLWHA	United States of America	37 people with HIV; 2 groups: Reiki + music vs. music only	Mixed-methods experiment; randomization to 2 groups; 6-week duration + 10-week follow-up	Group 1 received a Reiki session (by a trained practitioner) while listening to music; Group 2 only listened to music, without Reiki.	The Reiki + music group showed significant improvements in pain and stress; qualitative interviews supported reductions in anxiety and depression; the intervention was well-received and subjectively beneficial.
6	(Gregory et al. 2017)	Assessing the impact of massage therapy on anxiety, depression, hyperventilation, and quality of life in HIV patients	Belgium	29 adult HIV + patients; 15 massage, 14 control	4-week RCT; pre-post evaluation	Swedish massage therapy 1x/week (30 min, for 4 weeks), focusing on the back, performed by a professional physiotherapist	Massage caused a decrease in anxiety (HADS-A, *p* = 0.04) and hyperventilation (Nijmegen, *p* = 0.01); it had no significant impact on depression and the WHOQOL-HIV general quality of life domains, except for “Pain and discomfort” which improved.
7	(Garofalo et al. 2016)	Assessing the effectiveness of personalized daily text messages to improve ART adherence in HIV + adolescents and young adults	USA	105 participants aged 16–29 years, with low adherence to ART	Two-arm RCT, with 3, 6, and 12-month evaluations	Two-way, daily text messages, personalized to participant preferences, sent for 6 months	The intervention group was more likely to achieve ≥ 90% ART adherence (OR = 2.12, *p* < 0.05); the effect persisted for up to 12 months; satisfaction scores were very high.
8	(Martinez et al. 2018)	Testing the initial effectiveness of a couple-based HIV intervention (CLP) for Latino MSM men, integrating cultural and biomedical approaches.	USA	150 Latino male couples (*n* = 300 individuals)	3-arm RCT (intervention, waiting time, control), with 3 and 6 months follow-up	*Connecting Latinos en Pareja*(CLP): 4 couples sessions, focusing on PrEP, TasP, communication and decision-making skills, theory-based and culturally adapted	The study is still in protocol form; the primary outcomes to be measured are: the proportion of HIV-protected anal intercourse, as well as associated mediators/moderators.
9	(Jongbloed et al. 2016)	To evaluate a two-way mHealth-based text messaging intervention in reducing HIV risk in Indigenous youth who use drugs.	Canada	200 adolescents and young adults (14–30 years old) of Indigenous descent who use drugs	Zelen pre-randomized controlled trial (study protocol)	WelTel intervention: two-way weekly text messages, cell phone + plan, support from Cedar Project chaperones	The study is still in protocol form; it aims to reduce HIV risk, improve well-being, and improve access to services. Results are not yet available.
10	(Arnold et al. 2019)	To test the effectiveness of a culturally tailored behavioral intervention in reducing HIV risk behaviors in Black MSMW men.	USA	396 black men MSMW (men who have sex with men and women)	Randomized Controlled Trial, 9 months follow-up	4 individual counseling sessions + standard HIV testing and counseling	There were no significant differences between groups; both showed a decrease in unprotected sex. Short, culturally based counseling was quite effective.
11	(Bogart et al. 2023)	Assessing the effectiveness of the Rise intervention in improving ART adherence and reducing stigma	USA	166 Black/African American adults with HIV	Randomized Controlled Trial	MI-based counseling + social service referrals (housing, food)	Adherence increased significantly, stigma and mistrust decreased; no significant effect on viral suppression
12	(Carey et al. 2020)	Exploring the effectiveness of mindfulness training via telephone in improving ART & reducing risk	USA	42 PLWH, random to mindfulness vs. health coaching	Exploratory Randomized Clinical Trial	Mindfulness training vs. health coaching over the phone	There were no differences between groups; both showed increased adherence, decreased stress and sexual risk, and decreased impulsivity.
13	(Kuloor et al. 2019)	Assessing the effects of yoga on psychopathology and quality of life in people with HIV	India	60 HIV+ (30 yoga, 30 control)	Randomized Controlled Trial	8 weeks of intensive yoga (1 h/day, 5x/week): postures, breath, relaxation, meditation	Significant reduction in anxiety, depression, fatigue; significant improvement in quality of life compared to the control group
14	(Naoroibam et al. 2016)	Evaluating the impact of yoga on depression, anxiety, and CD4 count in HIV patients	India	44 HIV-1 patients (22 intervention, 22 control)	Randomized Controlled Pilot Study	1 month of Integrated Yoga (asana, pranayama, relaxation, meditation; 1 h/day)	Depression decreased significantly; CD4 increased significantly; anxiety did not differ significantly between groups.
15	(Harawa et al. 2020)	To develop and test Passport to Wellness (PtW), a client-centered intervention to increase engagement in HIV/STI prevention among Black MSM using incentives and peer support.	United States of America	80 eligible Black MSM.	Randomized controlled trial	This trial compared the full Passport to Wellness (PtW) intervention, which included peer support and incentives, with a version of the intervention that did not include the peer support component.	Significant increases in HIV and STI testing, as well as PrEP/PEP awareness and use, were observed among participants. However, no statistically significant differences were found between the groups with and without peer support.
16	(Harawa et al. 2018)	To test an intervention designed to reduce the frequency of condomless sex and the number of sex partners among recently incarcerated bisexual Black men.	United States of America	212 recently incarcerated bisexual black men.	Randomized controlled trial	Participants were randomly assigned to either a six-session small group intervention called Men in Life Environments (MILE) or a control group.	Both groups (intervention and control) showed significant reductions in risky behavior at the 3-month follow-up. The reduction in the intervention group was not significantly greater than in the control group.
17	(Rojas et al. 2025)	To adapt and test the efficacy of an online HIV/AIDS prevention intervention (HOMBRES de Familia) for Latino men, which also targets substance abuse and domestic violence.	United States of America	122 Latino men.	Randomized controlled trial	Participants were randomized to an intervention group that received four 1.5-hour video sessions (covering HIV prevention, substance use, and domestic violence through videos, role-playing, and skills training) or to a control group that received one video session on diabetes prevention.	This intervention was proven to be effective in increasing HIV knowledge, self-efficacy for prevention, and successfully reducing risky sexual behavior among participants in the intervention group.
18	(Pearson et al. 2020)	To test the efficacy of two culturally adapted counseling strategies as a means of HIV prevention among Native Americans, targeting symptoms of trauma or substance abuse.	United States of America	Native Americans 16 years of age and older.	Randomized comparative trial	Participants were randomized to receive one of two interventions: Narrative Exposure Therapy (NET), a trauma therapy focused on retelling memories, or Motivational Interviewing with Skills Training (MIST), a treatment to reduce substance use.	This document is a study protocol, so key findings regarding the efficacy of the interventions are not yet available. The study’s purpose was to determine the feasibility and efficacy of both interventions.

1. Culturally grounded messaging and materials
  Interventions were more acceptable and engaging when language, symbols, and narratives were tailored to participants’ lived cultures. In Zimbabwe, a CBT-based Life-Steps program was adapted with local idioms via the ADAPT-ITT framework and delivered by non-specialist counselors, yielding clear adherence gains to ART [20]. In the United States, a Spanish-language adaptation of the CARE plus computer counseling program aligned content and imagery with Latinx cultural references; participants rated it highly acceptable even though adherence and viral load did not significantly improve [21]. In India, yoga programs framed practices within familiar spiritual traditions, improving attendance, lowering depression and anxiety, and enhancing quality of life [22, 23]. Among Latino male couples, a culturally focused couple program integrated biomedical prevention alongside culturally attuned communication skills, formalized in a trial protocol [24]. An online family-centered program for Latino men integrated culturally relevant discussions of HIV, substance use, and family violence, increasing prevention knowledge and self-efficacy and reducing condomless sex (including sex under the influence of substances) [25]. Together, these studies show that precise adaptation of wording, imagery, and practices is central to cultural resonance and engagement.
2. Delivery through trusted peers and community spaces
  Who delivers the intervention and where it is delivered consistently shaped outcomes. The RISE program in the United States used peer counselors from Black communities to address stigma, medical mistrust, and structural barriers, improving adherence and reducing stigma and mistrust over time [26, 27]. In China, ChildCARE combined sessions for children, caregivers, and communities in familiar community settings, strengthening resilience, coping, hope, and emotional regulation when both child and caregiver components were offered [28]. Among Indigenous youth in Canada, the WelTel protocol paired weekly two-way text contact with Indigenous mentors and local chaperones to ensure cultural safety and trust in engagement [29]. Embedding programs within trusted peer networks and culturally safe spaces lowered barriers to participation and disclosure.
3. Integration of structural and practical supports
  Cultural adaptation was most consequential when paired with tangible supports that addressed real-world constraints. The extended RISE program combined motivational interviewing with referrals to housing and food resources, contributing to improved adherence and reduced medical mistrust [27]. ChildCARE integrated community activities that emphasized collective coping and practical support alongside skills training [28]. For Black MSM, Passport to Wellness added incentives and peer support to increase engagement in HIV and sexually transmitted infection prevention, boosting testing and awareness of PrEP and PEP even when differences between program variants were not statistically significant [30]. These approaches treat cultural fit and structural support as complementary, not competing.
4. Building resilience and coping capacities
  Many culturally adapted interventions emphasized resilience and coping as meaningful outcomes in their own right. ChildCARE’s multi-level design improved children’s resilience, coping, hope, and emotion regulation, particularly when both child and caregiver components were delivered together [28]. Telephone-delivered mindfulness training functioned as a flexible, culturally neutral modality that reduced stress, condomless sex, and sexual impulsivity and proved as feasible and acceptable as health coaching [31]. Yoga-based programs lowered anxiety, depression, and fatigue while improving quality of life and, in one pilot, increased CD4 T-cell counts [22, 23]. Complementary therapies further supported coping and symptom relief. Reiki delivered with music reduced pain and stress with qualitative reports of less anxiety and depression [32]. Short-course Swedish massage reduced anxiety and hyperventilation, and improved the pain-and-discomfort domain of WHOQOL-HIV (a validated quality-of-life measure for people living with HIV) [33]. These results indicate that strengthening coping capacities is a culturally meaningful pathway to impact beyond virological markers.
5. Flexible and accessible technology supports
  Digital tools were most effective when tailored to language, preferences, and cultural context. In the United States, daily personalized text messages for adolescents and young adults with low adherence increased the odds of achieving at least 90% adherence and were rated very highly by participants, with effects sustained up to 12 months [34]. The WelTel protocol demonstrated how weekly two-way texting could be embedded within Indigenous mentorship structures and local practices [29]. Mindfulness delivered by telephone offered a low-barrier option that matched the feasibility and acceptability of health coaching [31]. These studies show mHealth and eHealth are not “one size fits all,” but delivery channels to be culturally tuned.
6. Addressing specific sexual behaviours and prevention through cultural adaptation
  Culturally responsive counseling and group work showed mixed but informative effects on condomless sex and related prevention behaviours (for example, HIV testing intentions). Among Black MSMW in the United States, brief culturally tailored counseling reduced condomless sex across both intervention and control groups, suggesting even concise, culturally grounded content may shift behavior in real-world settings [35]. For recently incarcerated bisexual Black men, a six-session small-group program reduced risky behaviors at follow-up, again with similar reductions in control conditions [30]. Passport to Wellness increased HIV and STI testing and awareness and use of PrEP and PEP, although differences between full and pared-down versions were not statistically significant [36]. Among Native American communities, a protocol is comparing Narrative Exposure Therapy that targets trauma with Motivational Interviewing plus skills training addressing substance use; results will clarify culturally adapted pathways for prevention where trauma and substance use drive risk [37]. These studies, considered alongside culturally grounded couple and family programs for Latino communities [24, 25], underline both the promise and practical limits of culturally adapted prevention for key populations.
7. Effects on adherence and stigma as culturally mediated outcomes
  Adherence and stigma repeatedly emerged as outcomes that are culturally mediated by trust and relevance. Peer-delivered, community-embedded counseling in Black communities improved ART adherence and reduced stigma and medical mistrust [26, 27]. Locally adapted CBT in Zimbabwe improved adherence with high implementation fidelity by lay counselors [20]. Personalized texting strengthened adherence among youth and young adults, a group often underserved by traditional clinic-based adherence support [34]. Interventions that were acceptable and culturally resonant but did not shift clinical endpoints still offer value by building readiness and acceptability for future care steps, as seen with the Spanish-language computer counseling program guided by the Technology Acceptance Model [21].

## Discussion

This scoping review synthesized eighteen studies that examined culturally and contextually adapted interventions for people living with human immunodeficiency virus (HIV). The studies reflected a broad spectrum of approaches, including peer- and community-based counseling, digital health tools, mind-body therapies, and family- or couple-centered models. The overall pattern suggests that interventions are acceptable and feasible when they are tailored to the cultural, linguistic, and social contexts of the target population.

One reason culturally adapted materials resonated strongly in several studies is that language and symbolism are not just modes of communication but signals of belonging and respect. When counseling or behavioral content is translated into idioms and practices familiar to participants, it acknowledges lived realities and reduces the distance between intervention and everyday life. This mechanism helps explain why the adaptation of the Life-Steps cognitive behavioral therapy model in Zimbabwe was feasible and effective with lay counselors [20], while the Spanish-language CARE + program was highly acceptable even without measurable clinical impact [21]. Acceptability may thus precede and enable longer-term behavioral change.

Comparisons with prior research support this point. Earlier global HIV trials that used standardized materials without cultural tailoring often faced high attrition and low adherence [38, 39]. In contrast, culturally aligned interventions create an affective bond with participants that increases willingness to try and sustain new behaviors. However, the mixed clinical results, such as null changes in viral load in the CARE + study suggest that cultural fit alone may not be sufficient. It may act as a necessary foundation that requires reinforcement through structural or relational supports to yield clinical outcomes [40, 41].

Peer-delivered interventions appear effective because they reduce mistrust and stigma that are deeply rooted in historical and systemic inequities. The RISE studies [26, 27], showed that when counselors shared racial and community identity with participants, adherence improved and stigma declined. This reflects social identity theory, which posits that people are more receptive to guidance from in-group members. Similar findings have been documented in tuberculosis and substance use interventions, where peer educators improved retention by normalizing treatment [42, 43].

Community spaces also provided psychological safety. ChildCARE situated sessions within community centers, embedding HIV care in familiar environments and signaling that services belonged to, rather than intruded on, community life [28]. This contrasts with interventions delivered solely in clinical facilities, which previous studies identified as intimidating or alienating, particularly for marginalized populations [44, 45]. Taken together, these findings suggest that peer-led and community-located delivery mechanisms counteract stigma and create spaces of trust, a prerequisite for sustained engagement.

Structural supports (such as housing or food referrals) likely enhanced outcomes because they targeted upstream determinants of adherence. In the expanded RISE intervention, pairing motivational interviewing with tangible support reduced mistrust and improved adherence [27]. These findings align with earlier public health research showing that unmet basic needs consistently predict ART non-adherence [46]. When daily survival competes with medication routines, structural assistance can stabilize the environment so that behavioral change is feasible.

Comparative evidence illustrates this point further. Programs that relied solely on counseling or education without addressing social determinants often reported weaker or short-lived effects [47]. By contrast, interventions like Passport to Wellness demonstrated increased testing and prevention awareness even without significant between-arm differences, suggesting that combining psychosocial and material support may yield incremental benefits that are difficult to capture in short trial windows [30].

Interventions that emphasized resilience likely succeeded because they addressed the psychological burdens of stigma, poverty, and chronic illness. ChildCARE’s multi-level design supported coping, hope, and emotion regulation by mobilizing both children and caregivers [28]. This finding mirrors prior resilience research, which shows that multi-system interventions, targeting individual, family, and community produce more durable effects in children facing adversity [48]. By contrast, interventions that focused narrowly on individuals often saw benefits dissipate over time.

Complementary therapies such as yoga, Reiki, and massage add another layer of resilience by integrating mind-body practices rooted in cultural traditions. Their effects on anxiety, stress, and quality of life [22, 23, 32, 33] echo findings from psycho-oncology, where such practices improved symptom management and emotional regulation [49, 50]. The appeal of these therapies may stem from their alignment with local cultural values of balance and holistic well-being, which conventional biomedical approaches often overlook.

Technology-based interventions improved adherence in part because they reduced barriers of distance, stigma, and time. Personalized daily text messaging helped adolescents and young adults maintain adherence by embedding reminders into daily routines [34]. Unlike one-way reminders tested in earlier adherence trials, these personalized and interactive texts established relational accountability, which participants valued [51, 52].

However, technology also risks reinforcing inequities. The WelTel protocol for Indigenous youth combined two-way messaging with mentorship to mitigate alienation from formal health systems [29]. This approach contrasts with earlier mobile interventions that were purely informational, which often showed limited impact in marginalized groups [53]. The comparison suggests that digital platforms are most effective when combined with cultural grounding and reciprocal communication, rather than being used as stand-alone technological fixes.

Family and couple approaches may be effective because they leverage relational dynamics to sustain behavior change. ChildCARE demonstrated stronger effects when caregiver and child components were combined [28], reflecting family systems theory, which holds that change in one member is reinforced when others in the system adopt parallel strategies [54]. Similarly, the couple-based protocol for Latino men who have sex with men sought to integrate communication and shared decision-making into prevention, building on evidence that dyadic interventions can improve safer sex negotiation [33].

Comparisons with previous literature underscore a persistent gap in evaluations that directly contrast culturally adapted versus non-adapted interventions, limiting causal inference about the added value of adaptation. Most earlier HIV interventions treated individuals as isolated units, neglecting relational dynamics [55]. Studies among recently incarcerated men and men who have sex with men and women showed risk reductions across groups but not specifically in the intervention arm [30, 35]. This suggests that while relational contexts influence risk, interventions need more precise tailoring to partnership structures and post-incarceration challenges to show distinct effects.

### Limits and future directions

This is a scoping review that maps the breadth of evidence, so we did not conduct a formal quality appraisal or risk of bias assessment under PRISMA ScR guidance; as a result, certainty of evidence cannot be inferred. Marked heterogeneity in interventions, comparators, outcomes, and follow up precluded a meta analysis, and our synthesis is descriptive. The search was limited to English and selected databases, and we did not systematically search grey literature or contact experts; despite a two tier strategy and a sensitivity step that removed cultural terms, some studies may have been missed. Several included records were study protocols without outcomes, and generalizability is constrained by uneven geography and short follow up in some studies. The body of evidence shows variability. Some interventions produced psychosocial gains without clinical outcomes [21, 31], and three studies remain protocols without published results [24, 29, 37]. This reinforces the need for caution in interpretation. Future research should use adequately powered designs across diverse cultural contexts, explicitly measure long-term sustainability, and include economic and implementation outcomes to assess scalability.

## Conclusion

This scoping review suggests that contextually and culturally tailored interventions for people living with human immunodeficiency virus were often associated with improved engagement and psychosocial outcomes. Across the reviewed studies, many programs incorporated cultural and linguistic alignment, delivery by trusted peers in community settings, complementary mind body strategies, and technology enabled contact. Effects on clinical endpoints such as virological suppression were mixed and some trials reported null findings, while several interventions were available only as protocols. These features indicate that the current evidence base maps promising directions rather than confirming causal effects.

The implications for policy and practice are to prioritize approaches that are acceptable and feasible in local contexts. Programs can consider the specific needs of adolescents, women, Indigenous communities, and sexual minorities rather than assuming a universal model. Interventions that combine antiretroviral therapy support with psychosocial capacity building, relational support within families and partnerships, and practical assistance may enhance participation where structural barriers and stigma are salient. Mobile health and electronic health platforms can extend reach when adapted to local language and norms and when paired with involvement of local actors such as peer counselors and caregivers.

Future research should use adequately powered evaluations in diverse settings, report durability beyond initial follow up, and include economic and implementation outcomes that speak to feasibility and scale. Development and testing of hybrid models that combine technology, community delivery, and culture based psychosocial strategies would be valuable. Incorporating community based participatory research into design can help ensure local relevance and sustainability. Finally, more detailed reporting of adaptation processes through protocols and implementation studies would support transparent replication and informed adaptation across contexts.

## Supplementary Information

Below is the link to the electronic supplementary material.

Supplementary Material 1

## Data Availability

All data generated or analysed during this study are included in this published article.
