# Supplementary material for: Contextual and culturally adapted interventions to improve HIV outcomes: a scoping review
Source: BMC Infect Dis. 2025 Dec 29;25:1760. doi: 10.1186/s12879-025-12143-3 (PMC12752318; doi:10.1186/s12879-025-12143-3)
Supplement: Supplementary file 1 — Supplementary Material 1 [file 12879_2025_12143_MOESM1_ESM.docx]

**Appendix 1.** Searching Strategy

| **Database** | **Keywords** |
| --- | --- |
| **PubMed** | ("HIV"[MeSH Terms]) OR ("HIV"[Text Word])) OR ("HIV"[Title/Abstract])) OR ("HIV infection"[Text Word])) OR ("HIV infection"[Title/Abstract])) OR ("HIV prevention"[Text Word])) OR ("HIV prevention"[Title/Abstract])) OR ("HIV treatment"[Text Word])) OR ("HIV treatment"[Title/Abstract])) OR ("AIDS"[Text Word])) OR ("AIDS"[Title/Abstract]) |
|  | ("intervention"[Text Word]) OR ("intervention"[Title/Abstract]) OR ("program"[Text Word]) OR ("program"[Title/Abstract]) OR ("approach"[Text Word]) OR ("approach"[Title/Abstract]) OR ("trial"[Text Word]) OR ("trial"[Title/Abstract]) OR ("evaluation"[Text Word]) OR ("evaluation"[Title/Abstract]) OR ("implementation"[Text Word]) OR ("implementation"[Title/Abstract]) OR ("service"[Text Word]) OR ("service"[Title/Abstract]) OR ("delivery"[Text Word]) OR ("delivery"[Title/Abstract]) OR ("model"[Text Word]) OR ("model"[Title/Abstract]) |
|  | ("cultural adaptation"[Text Word]) OR ("cultural adaptation"[Title/Abstract]) OR ("culturally adapted"[Text Word]) OR ("culturally adapted"[Title/Abstract]) OR ("cultural tailoring"[Text Word]) OR ("cultural tailoring"[Title/Abstract]) OR ("cultural competence"[MeSH Terms]) OR ("cultural competence"[Title/Abstract]) OR ("cultural safety"[Text Word]) OR ("cultural safety"[Title/Abstract]) OR ("Indigenous led"[Title/Abstract]) OR ("community led"[Title/Abstract]) OR ("community driven"[Title/Abstract]) OR ("community based"[Title/Abstract]) OR ("peer led"[Title/Abstract]) OR ("context specific"[Title/Abstract]) OR ("locally adapted"[Title/Abstract]) OR ("place based"[Title/Abstract]) OR ("structural intervention"[Title/Abstract]) OR ("stigma reduction"[Title/Abstract]) OR ("adherence support"[Title/Abstract]) OR ("differentiated service delivery"[Title/Abstract]) |
|  | (adherence[Title/Abstract]) OR (stigma[Title/Abstract]) OR ("engagement in care"[Title/Abstract]) OR (retention[Title/Abstract]) OR (linkage[Title/Abstract]) OR ("viral suppression"[Title/Abstract]) OR ("quality of life"[Title/Abstract]) |
| **Scopus** | TITLE-ABS-KEY ( HIV ) OR TITLE-ABS-KEY ( "HIV infection" ) OR TITLE-ABS-KEY ( "HIV prevention" ) OR TITLE-ABS-KEY ( "HIV treatment" ) OR TITLE-ABS-KEY ( AIDS ) |
|  | TITLE-ABS-KEY ( intervention* ) OR TITLE-ABS-KEY ( program* ) OR TITLE-ABS-KEY ( approach* ) OR TITLE-ABS-KEY ( trial* ) OR TITLE-ABS-KEY ( evaluation* ) OR TITLE-ABS-KEY ( implementation* ) OR TITLE-ABS-KEY ( service* ) OR TITLE-ABS-KEY ( deliver* ) OR TITLE-ABS-KEY ( model* ) |
|  | TITLE-ABS-KEY ( "cultural adaptation" ) OR TITLE-ABS-KEY ( "culturally adapted" ) OR TITLE-ABS-KEY ( "cultural tailoring" ) OR TITLE-ABS-KEY ( "cultural competence" ) OR TITLE-ABS-KEY ( "cultural safety" ) OR TITLE-ABS-KEY ( "Indigenous led" ) OR TITLE-ABS-KEY ( "community led" ) OR TITLE-ABS-KEY ( "community driven" ) OR TITLE-ABS-KEY ( "community based" ) OR TITLE-ABS-KEY ( "peer led" ) OR TITLE-ABS-KEY ( "context specific" ) OR TITLE-ABS-KEY ( "locally adapted" ) OR TITLE-ABS-KEY ( "place based" ) OR TITLE-ABS-KEY ( "structural intervention" ) OR TITLE-ABS-KEY ( "stigma reduction" ) OR TITLE-ABS-KEY ( "adherence support" ) OR TITLE-ABS-KEY ( "differentiated service delivery" ) |
|  | TITLE-ABS-KEY ( adherence ) OR TITLE-ABS-KEY ( stigma ) OR TITLE-ABS-KEY ( "engagement in care" ) OR TITLE-ABS-KEY ( retention ) OR TITLE-ABS-KEY ( linkage ) OR TITLE-ABS-KEY ( "viral suppression" ) OR TITLE-ABS-KEY ( "quality of life" ) |
| **CINAHL** | (MH "HIV Infections+") OR TX(adherence OR "HIV infection" OR "HIV prevention" OR "HIV treatment" OR AIDS) |
|  | (MH "Intervention Studies+") OR TX(intervention* OR program* OR approach* OR trial* OR evaluation* OR implementation* OR service* OR deliver* OR model*) |
|  | (MH "Cultural Competence+") OR TX("cultural adaptation" OR "culturally adapted" OR "cultural tailoring" OR "cultural safety" OR "Indigenous led" OR "community led" OR "community driven" OR "community based" OR "peer led" OR "context specific" OR "locally adapted" OR "place based" OR "structural intervention" OR "stigma reduction" OR "adherence support" OR "differentiated service delivery") |
|  | (MH "Patient Compliance+") OR MH "Quality of Life+" OR TX(adherence OR stigma OR "engagement in care" OR retention OR linkage OR "viral suppression" OR "quality of life") |
